# Self-Harm Intervention: Family Therapy (SHIFT), a study protocol for a randomised controlled trial of family therapy versus treatment as usual for young people seen after a second or subsequent episode of self-harm  

**DOI:** 10.1186/s13063-015-1007-4

**Published:** 2015-11-04

**Authors:** Alexandra Wright-Hughes, Elizabeth Graham, Amanda Farrin, Michelle Collinson, Paula Boston, Ivan Eisler, Sarah Fortune, Jonathan Green, Allan House, David Owens, Mima Simic, Sandy Tubeuf, Jane Nixon, Christopher McCabe, Michael Kerfoot, David Cottrell

**Affiliations:** Leeds Institute of Clinical Trials Research, University of Leeds, Leeds, UK; Leeds Institute of Health Sciences, University of Leeds, 101 Clarendon Road, LS2 9LJ Leeds, UK; Institute of Psychiatry, King’s College London, London, UK; Academic Department of Child and Adolescent Psychiatry, University of Manchester, Manchester, UK; Faculty of Medicine and Dentistry, University of Alberta, Alberta, Canada

**Keywords:** Self-harm, suicide, young people, child and adolescent mental health, family therapy, randomised controlled trial

## Abstract

**Background:**

Self-harm is common in the community with a lifetime prevalence of 13 %. It is associated with an elevated risk of overall mortality and suicide. People who harm themselves are high users of public services. Estimates of the 1-year risk of repetition vary between 5 and 15 % per year. Currently, limited evidence exists on the effectiveness of clinical interventions for young people who engage in self-harm. Recent reviews have failed to demonstrate any effect on reducing repetition of self-harm among adolescents receiving a range of treatment approaches. Family factors are particularly important risk factors associated with fatal and non-fatal self-harm among children and adolescents. Family therapy focuses on the relationships, roles and communication patterns between family members, but there have been relatively few studies of specifically family-focused interventions with this population. The Self-Harm Intervention: Family Therapy (SHIFT) Trial was funded by the National Institute for Health Research (NIHR) Health Technology Assessment programme (grant no. 07/33/01) following a commissioned call for this research.

**Methods/Design:**

SHIFT is a pragmatic, phase III, multicentre, individually randomised, controlled trial comparing Family Therapy (FT) with treatment as usual (TAU) for adolescents aged 11 to 17 who have engaged in at least two episodes of self-harm. Both therapeutic interventions were delivered within the National Health Service (NHS) Child and Adolescent Mental Health Services (CAMHS) in England. Participants and therapists were, of necessity, aware of treatment allocation, but the researchers were blind to the allocations to allow unbiased collection of follow-up data. Primary outcome data (repetition of self-harm leading to hospital attendance 18 months post-randomisation) were collected from the Health and Social Care Information Centre (HSCIC), augmented by directed searches of medical records at Acute Trusts. Secondary outcome data (including suicidal intent, depression, hopelessness and health economics) were collected at 12 and 18 months post-randomisation via researcher-participant interviews and by post at 3 and 6 months.

**Discussion:**

SHIFT will provide a well-powered evaluation of the clinical and cost effectiveness of Family Therapy for young people who have self-harmed on more than one occasion. The study will be reported in 2016, and the results will inform clinical practice thereafter.

**Trial registration:**

ISRCTN59793150. 26 January 2009.

## Background

The SHIFT Trial protocol was developed in response to a commissioned call for research. In accordance with the commissioning brief and in line with current UK clinical practice, self-harm is defined as any form of non-fatal self-poisoning or self-injury (such as cutting, taking an overdose, hanging, self-strangulation, jumping from a height, and running into traffic), regardless of motivation or the degree of intention to die. This definition includes what in the United States would be described as non-suicidal self-injury.

Self-harm is common in the community; a systematic review suggested a lifetime prevalence of self-harm of 13 % [1], whilst a recent meta-analysis reported an adjusted lifetime prevalence of non-suicidal self-injury of 17 % [2]. Self-harm is associated with an elevated risk of overall mortality [3–6] and suicide. In one follow-up study [7] of 15- to 24-year olds, who had presented to hospital following an episode of self-harm, the overall number of deaths from all causes was 3 % of cases, four times higher than expected. This was mainly due to an excess number of suicides (2 %), which were 10 times more frequent than expected. A recent multicentre study of those under 18 presenting following self-harm found that while mortality was relatively low at 0.9 % at a median follow-up period of 6 years, nearly half of these deaths were due to suicide [8]. Due to the young ages at which these deaths occur, the life years lost to the community due to suicide and the impact on family members are considerable.

Around 20–30,000 adolescents present to hospital each year having harmed themselves [9] although this represents only one in eight episodes of self-harm because the majority of young people who self-harm do not present to hospital [10]. Rates of self-harm amongst young people in the UK are not declining [11], and the epidemiology of self-harm appears to be shifting with dangerous methods such as hanging increasing in females [12], with higher rates among South Asian young women [13]. People who harm themselves are high users of public services [14], and increasing rates of self-harm will lead to an even greater demand for services.

In studies based on presentations to general hospitals in the UK, the majority of adolescents have harmed themselves by taking an overdose; self-poisoning with analgesics is particularly common [11, 15] and dangerous due to the risk of death from liver failure. At the community level, the most common methods of self-harm are cutting and overdose [10]. Young people have a poor understanding of the potential lethality of methods of self-harm, so interventions to prevent further episodes of self-harm is one approach to reducing both the morbidity and mortality associated with these acts.

Estimates of risk of 1-year repetition vary between 5 and 15 % per year [16], with 18 % in a recent UK multicentre monitoring study of over 5000 adolescents [11] and as much as 27 % in an average of 5 years of follow-up of around 4000 adolescents [8]. In addition, actual rates may be much higher if repetition that does not come to clinical attention is considered [9]. The risk of repetition is highest in the first year, but may remain high for many years after an episode [4, 17].

Currently, there is limited evidence about the effectiveness of clinical interventions for young people who engage in self-harm, although there has been much work in the area since the earliest systematic review [18]. Recent reviews have failed to demonstrate any effect on reducing repetition of self-harm among adolescents receiving a range of treatment approaches including therapeutic assessments and compliance enhancement in hospitals, youth-nominated support teams, tokens for hospital admission and home and family-focused interventions [19, 20]. The early promise of developmental group psychotherapy has not been replicated in larger studies [20]. Brent et al. [21] focused on aspects of trials that showed promise and concluded that interventions that activated family support, addressed motivations for change, were quickly mobilised (to reflect the elevated risk of repetition immediately post-episode) and promoted a positive affect are most likely to be able to demonstrate effectiveness. The heterogeneity of treatment as usual (TAU) and the lack of its characterisation of TAU [21], in addition to the many underpowered studies, have held back advances in this field.

Family factors are particularly important risk factors associated with fatal and non-fatal self-harm among children and adolescents [20, 22–25]. Difficulties in parent–child relationships, including those related to early attachment problems, and perceived low levels of parental caring and communication are related to increased risk of suicide and self-harm among children and adolescents [26]. A family history of self-harm is associated in adolescents with increased risk for suicide deaths [27–29] and for non-fatal self-harm [10, 30]. Parental mental illness and substance abuse are significant risk factors [16]. A strong association exists between self-harm and both childhood sexual abuse and physical abuse [31]. Young people who self-harm experience higher rates of exposure to recent stressful life events such as rejection, conflict or loss following the break-up of a relationship, conflicts and disciplinary or legal crises [15].

Systemic family therapy focuses on mobilising family resources and on the relationships, roles and communication patterns between family members. Surprisingly, there is only a small amount of literature on the use of family therapy with young people who self-harm [32–34], with most studies not powered to detect reductions in repeat self-harm. The SHIFT study seeks to evaluate the potential clinical and cost effectiveness of a family therapeutic intervention following adolescent self-harm.

## Methods/Design

### Design

**Fig. 1:**
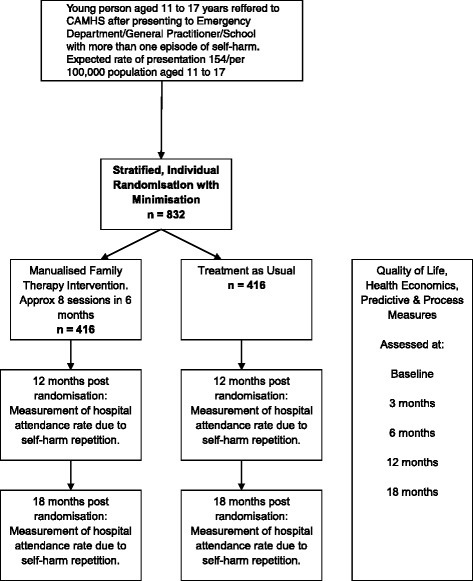
Trial design flow diagram

SHIFT is a pragmatic, phase III, multicentre, individually randomised, controlled trial comparing Family Therapy (FT) with treatment as usual (TAU) for adolescents aged 11 to 17 who have engaged in at least two episodes of self-harm. An individually randomised design (Fig. 1) was chosen over a cluster randomised design as the risk of contamination between the two treatment arms was minimal: participants attended appointments at varying times and so could not cross compare, and family therapists were specifically instructed not to treat any participants in the TAU arm of the trial wherever possible.

### Trial objectives and outcomes

The primary objective of the SHIFT trial is to assess the effectiveness of FT compared to TAU as measured by rates of repetition of self-harm leading to hospital attendance 18 months after randomisation. This primary outcome was selected because it is an objective measure of self-harm. This outcome can be quantified using routine hospital records data rather than relying on participant self-report and can be obtained even if contact has been lost with participants, thereby maximising the accuracy and completeness of the data collection.

Secondary objectives are to assess the following:

1. The effectiveness of FT compared to TAU, measured by repetition rates of self-harm leading to hospital attendance at 12 months after randomisation.
2. The cost per self-harm event avoided due to FT, measured using a structured health economics questionnaire designed by the trial health economist to be specific to this population.
3. The characteristics of further episodes of self-harm (all episodes, not just those resulting in hospital attendance). This includes the number of subsequent self-harm events, time to next event, severity of event (fatal, near fatal or not) and dangerousness of the method used, as measured by the Suicide Attempt Self-Injury Interview (SASII) [35].
4. Suicidal ideation in each trial arm, measured by the Beck Scale for Suicide Ideation [36].
5. Quality of life in each arm, measured by the Paediatric quality of Life Enjoyment and Satisfaction measure (PQ-LES) [37] and parental completion of the General Health Questionnaire-12 (GHQ-12) [38].
6. Depression in each arm, measured by the Children’s Depression Rating Scale – Revised (CDRS-R) [39].
7. Overall mental health and emotional and behavioural difficulties in each arm via young person and parental completion of the Strengths and Difficulties Questionnaire (SDQ) [40].
8. Hopelessness in each arm, via completion of the Hopelessness Scale for Children [41].
9. Family functioning in each arm, measured by the McMaster Family Assessment Device [42] and the Family Questionnaire [43].
10.  Mediator and moderator variables that influence engagement with and benefit from treatment (for example, number of sessions, medication use, referrals, mediators).
11. Therapeutic alliance to family therapy, via family therapist, young person and parental completion of the System for Observing Family Therapy Alliances (SOFTA) [44].
12.  Therapist adherence to the family therapy manual. A trial-specific approach has been developed for independent review of a representative sample of recorded FT sessions.

### Recruitment setting and participants

Young people and their primary caregivers were identified from 40 National Health Service (NHS) Child and Adolescent Mental Health Services (CAMHS) across three ‘hubs’ in England - Greater Manchester, London and Yorkshire. Following consultation with service users and clinicians, we elected not to screen young people at first presentation in the emergency department or primary care because in this time of crisis, immediately following the self-harm event, young people and their families would not be in a position to readily give fully informed consent. Young people were therefore screened for trial suitability at their first presentation to CAMHS following self-harm. They would thus have already been assessed by the emergency department staff, on-call CAMHS staff in hospital or by primary/community health staff before referral to CAMHS, where the screening took place. To be included in the trial, participants were required to meet the eligibility criteria described next.

### Inclusion criteria

Inclusion criteria included the following:

1. Aged 11–17 years.
2. Self-harmed prior to assessment by the CAMHS team (self-harm being the key feature of presentation).
3. Engaged in at least one previous episode of self-harm (recorded by CAMHS or via self-report) prior to the index presentation.
4. Where the presenting episode was due to alcohol or recreational drugs, the young person had explicitly stated that he / she was intending self-harm by use of these substances.
5. The clinical intention was to offer CAMHS follow-up for self-harm.
6. Lived with primary care-giver.
7. Both young person and primary care-giver gave written informed consent, as appropriate.

### Exclusion criteria

Exclusion criteria include the following:

1. At serious risk of suicide (clinical judgement);
2. An ongoing child protection investigation within the family, which would have made treatment difficult to deliver;
3. Would not have ordinarily been treated in generic CAMHS but rather by a specific service (for example, psychiatric inpatient care for severe major depressive disorder);
4. Pregnant at time of trial entry;
5. Actively being treated in CAMHS (as the possibility of randomisation might disrupt ongoing therapy);
6. In a children’s home or short-term foster placement;
7. Moderate to severe learning disability or lacked capacity to comply with trial requirements;
8. Involved in another research project - at the time of trial entry or within the last six months;
9. Sibling had been randomised to the SHIFT trial, or was receiving family therapy within CAMHS; or
10. The young person and one main care-giver had insufficient proficiency in English to contribute to the data collection.

All young people presenting to CAMHS following self-harm were screened. Data were recorded anonymously for those not meeting the above criteria in order to monitor trial uptake and representativeness of the trial population. Where a young person met the criteria, the assessing CAMHS clinician discussed trial participation at the assessment appointment, passed on the participant information sheets and requested consent for subsequent contact by a researcher. Those providing such consent were contacted by the researcher, who arranged to visit the family at home, obtained consent for participation in the trial and administered the baseline assessment.

### Recruitment, randomisation and blinding

Where the appropriate consent was obtained and baseline assessment completed, the researchers randomised participants sequentially via an automated system at the Clinical Trials Research Unit (CTRU) at the University of Leeds. Participants were randomly allocated on a 1:1 basis to receive FT or TAU, using a computer-generated minimisation programme incorporating a random element to ensure the treatment arms were well balanced for the following: centre (CAMHS team), gender, age (11 to 14 or 15 to 17), living arrangements (with parents or guardians/foster care), number of previous self-harm episodes (including index event) (2 or ≥ 3), and type of index episode (self-poisoning, self-injury, or combination). Where therapists were not aligned to a specific service but covered a number of services, additional randomisation of the lead therapist took place within the FT team in order to minimise case-selection bias.

Participants and therapists were, of necessity, aware of treatment allocation, but researchers were blind to the allocation to allow unbiased collection of follow-up data. CTRU was responsible for informing CAMHS clinicians and family therapists of randomisation outcome, to maintain researcher blinding.

### Data collection

Required data, assessment tools, collection time points and processes are summarised in Table 1.

**Table 1:** Summary of assessments

Assessment (including who is involved)	Timeline (months post-randomisation)				
	Baseline	3	6	12	18
Eligibility and consent					
- Eligibility (assessed by clinician)	X				
- Consent (YP, P, R^a^)	X				
Background and demographics (YP, P, R - interview and case notes)					
- Personal details	X				
- Outline ‘index’ event details	X				
- Current co-morbid physical/mental health	X				
- Current psychotropic medications	X				
- History of abuse	X				
Follow-up data (collected from case notes)					
- Therapy details (provided by therapist)			X	X	
- Therapist supervision details (provided by therapist/supervisor)			X	X	
- Details of further self-harm episodes since consent (R)				X	X
- Psychotropic medication details (R)				X	X
- Referrals to other MH services (R)				X	X
- Re-referral to CAMHS (R)				X	X
- Admissions to hospital relating to mental health (R)				X	X
- All-cause mortality (CTRU to collect via MRIS flagging)					X
- Serious adverse event reporting and hospital attendance (R and HSCIC)	Ongoing collection				
Questionnaires (completed at researcher visit unless otherwise stated)					
- Family Questionnaire (P self-report, CTRU postal admin at 3 and 6 months)	X	X	X		
- System for Observing Family Therapy Alliances - SOFTA (completed by the family therapist and participants at Family Therapy session 3)		X			
- Suicide Attempt Self-Injury Interview - SASII (Interview with YP)	X			X	X
- Beck Scale for Suicide Ideation (YP self-report)	X			X	X
- Hopelessness Scale for Children (YP self-report)	X			X	X
- McMaster Family Assessment Device – FAD (YP and P self-report)	X			X	X
- General Health Questionnaire 12 – GHQ (P self-report)	X			X	X
- Strengths and Difficulties Questionnaire - SDQ (YP and P self-report)	X			X	X
- Children’s Depression Rating Scale – CDRS (Interview with YP)	X			X	X
- Paediatric Quality of Life Enjoyment and Satisfaction – PQ-LES (YP self-report)	X			X	X
- Inventory of Callous Unemotional Traits - ICU (YP self-report)	X				
- EQ-5D (YP self-report, CTRU postal admin at 6 months)	X		X	X	X
- Health Utilities Index 3 – HUI3 (P self-report, CTRU postal admin at 6 months)	X		X	X	X
- Health Economics questionnaire (YP and P self-report, CTRU postal admin at 3 and 6 months)	X	X	X	X	X

^a^YP, young person; P, parent/care-giver; R, researcher; HSCIC, NHS Health and Social Care Information Centre

### Baseline

At the baseline assessment, the researcher administered questionnaires to the young person, and self-reported questionnaires were also completed by the young person and the consenting primary caregiver.

### Follow-up

The primary outcome measure was obtained from Accident and Emergency Departments (A&E) and in-patient Hospital Episode Statistic (HES) data downloads from the NHS Health and Social Care Information Centre (HSCIC), which holds hospital attendance and admission data for hospitals across England. This method of primary outcome data collection was augmented by directed hospital record searches, undertaken by researchers at frequent intervals throughout the trial. Researchers searched Acute Trust records for episode details that were unclear from the central HES data, or for any hospital attendances for those participants who had not consented to the trial team providing their details to the HSCIC. Given that HES data sets are England-wide, this maximised collection of hospital attendance data, allowing for participants moving out of catchment area (within England). Collection of these routine data also minimised bias by eliminating the possibility of preferential researcher data collection at certain Acute Trusts.

Postal questionnaires were administered by CTRU at 3 and 6 months post-randomisation. If questionnaires were not returned, postal reminders were sent 2 weeks after the initial mailing and then again a further 2 weeks later.

Secondary outcome were obtained via face-to-face researcher interviews at 12 and 18 months following randomisation.

Data relating to treatment were provided by the treating CAMHS clinicians or Family Therapists, Clinical Studies Officers (CSOs) employed by the Research Networks in each locality and by the researchers, when blinding was no longer required (that is, after participant-reported follow-up data had been collected). The data included session attendance (FT and TAU), therapeutic approach (for TAU), referrals within and outside CAMHS, requirements for psychotropic medications, and liaison with other agencies. In addition, treating clinicians and participants completed the System for Observing Family Therapy Alliances scale (SOFTA) [44] at the end of the third treatment session. Supervision sessions for Family Therapists, and routinely provided clinical supervision for CAMHS clinicians were also recorded.

### Intervention

Both therapeutic interventions were delivered within CAMHS, and all participants were treated within their local service. Family therapists were formally linked with specific CAMHS teams to ensure lines of clinical responsibility were clear, and all clinicians in both arms of the trial had access to local child and adolescent psychiatrists if medication or hospitalisation needed to be considered.

### Family therapy

The Family Therapy intervention was based on a modified version of the Leeds Family Therapy & Research Centre Systemic Family Therapy Manual (LFTRC Manual), the development and validation of which was funded by the Medical Research Council (MRC)  to support trials of FT [45]. This manual was updated by the Family Therapy expert members from the Trial Management Group to ensure it was appropriate for work with families following self-harm. Qualified Family Therapists (those eligible for registration with the UK Council for Psychotherapy - UKCP) were appointed specifically to work on the trial. Family therapists worked in teams of 3 or 4 and provided trial FT as a team for a cluster of CAMH services. They received initial training, undertook pilot work and received monthly supervision from the SHIFT Family Therapy leads in each locality. Annual meetings of the trial therapists were organised to ensure ongoing consistency of the treatment approach across all trial sites.

Plans were made for the young people and their families to attend FT sessions of approximately 1¼ hours duration each, delivered over 6 months at approximately monthly intervals but with more frequent initial appointments. This equated to approximately 8 sessions, but there was the expectation that some participants would receive fewer sessions due to drop-out or mutually agreed-upon termination of treatment. Equally, it was anticipated that some might receive more sessions (within the predefined 6-month period, or extending beyond 6 months) where this was deemed clinically appropriate.

Wherever possible, and where consent was provided, sessions were video recorded as this is part of good family therapy practice and facilitates supervision. In addition, this procedure facilitated central review of a selection of sessions to monitor adherence to the manual and allow reporting of this.

### Treatment as usual

TAU was the care offered by local CAMHS teams to adolescents referred following self-harm. This treatment was anticipated to be diverse and involve individual and/or family-orientated work, delivered by a range of practitioners with various theoretical orientations. As SHIFT is a pragmatic trial involving a number of collaborating CAMHS teams, the specification of TAU was not deemed possible or appropriate, although it was expected that CAMHS practitioners would be working in line with best practice as set out in several National Institute for Health and Care Excellence (NICE) guidelines (for example, guidance on self-harm and depression in childhood [46, 47]). TAU thus involved a wide range of treatment techniques and modalities (such as supportive counselling or cognitive behaviour therapy) that were not delivered to the FT group as part of the clinical intervention, unless they became indicated during or after family therapy.

### Contamination

The possibility of cross-arm contamination was considered during the design stage of the trial, with the following points noted and action taken:

1. Different teams of therapists delivered the two interventions in each CAMH service, and the SHIFT family therapists were prohibited (wherever possible) from treating participants in the TAU arm for the duration of the trial. However, in the few cases where this was unavoidable, contamination was recorded and levels will be reported.
2. Due to the nature of appointment scheduling, and the fact that this was family-specific therapy (that is, not a group intervention), there was little opportunity for participants to meet and discuss treatment, so contamination was very unlikely.
3. Any family-orientated clinical interventions in the TAU group were likely to be different from the trial FT intervention, which required adherence to the LFTRC manual, fully-trained family therapists eligible for United Kingdom Council for Psychotherapy (UKCP) registration, therapy delivered in a team context and regular supervision.

### Safety monitoring

Non-serious adverse events were operationally defined in SHIFT as treatment on an emergency outpatient basis (A&E attendance) and re-referral to CAMHS, as these events are expected to occur within the adolescent study population. Deaths and hospital admissions were defined as serious adverse events because, although not as common, they are to be expected in the population being studied.

An independent Data Monitoring and Ethics Committee (DMEC) is scheduled to meet at least annually to review the safety and ethics of the trial during recruitment and follow-up. The DMEC review the number and frequency of hospitalisations and deaths overall and as a consequence of self-harm and could recommend trial suspension or closure if a significantly increased frequency of such events in one or both arms is identified.

### Research governance

The Trial is managed on a day-to-day basis by the Chief Investigator and a core team from the Clinical Trials Research Unit at the University of Leeds. All collaborators, including trial research staff, form the Trial Management Group, which meets on a regular basis to review progress. Independent oversight is provided by the Trial Steering Committee and Data Monitoring and Ethics Committee.

The trial was reviewed and approved by the NHS Research Ethics Committee (REC)  Yorkshire and the Humber - Leeds West (REC reference 09/H1307/20). SHIFT also received R&D approval from all participating CAMHS’ host organisations. For the purposes of collecting routine data from Acute Trusts to inform the primary outcome, the REC agreed that the study was exempt from Site Specific Assessment, but Researchers required Letters of Access from each Trust to access records. Participants could withdraw from further researcher contact, receipt of postal questionnaires or collection of clinical data from CAMHS/Acute Trusts/HSCIC. Withdrawal requests were respected throughout the trial and data were only obtained where valid consent remained in place.

The following organisations gave research governance approval for the study:

5 Boroughs Partnership NHS Foundation Trust

Airedale NHS Foundation Trust

Alder Hey Children’s NHS Foundation Trust

Barnet and Chase Farm Hospitals NHS Trust

Barnet, Enfield and Haringey Mental Health NHS Trust

Bolton NHS Foundation Trust

Bradford District Care NHS Foundation Trust

Bradford Teaching Hospitals NHS Foundation Trust

Calderdale & Huddersfield NHS Foundation Trust

Central Manchester University Hospitals NHS Foundation Trust

Croydon Health Services NHS Trust (formerly Mayday)

Dartford and Gravesham NHS Trust

East London NHS Foundation Trust

Guy’s and St Thomas’ NHS Foundation Trust

Harrogate and District NHS Foundation Trust

Homerton University Hospital NHS Foundation Trust

Hull and East Yorkshire Hospitals NHS Trust

Humber NHS Foundation Trust

Kings College Hospital NHS Foundation Trust

Leeds Community Healthcare NHS Trust

Leeds Teaching Hospitals NHS Trust

Leeds and York Partnership NHS Foundation Trust

Lewisham Healthcare NHS Trust

The Mid Yorkshire NHS Trust

North Lincolnshire and Goole Hospitals NHS Foundation Trust

North Middlesex University Hospital NHS Trust

Oxleas NHS Foundation Trust

Pennine Acute Hospitals NHS Trust

Pennine Care NHS Foundation Trust

Royal Free Hampstead NHS Trust

St George’s Healthcare NHS Trust

St Helens and Knowsley Teaching Hospitals NHS Trust

Salford Royal NHS Foundation Trust

Scarborough and NE Yorkshire Healthcare NHS Trust

South London and Maudsley NHS Foundation Trust

South London Healthcare NHS Trust

South Tees Hospitals NHS Foundation Trust

South West Yorkshire Partnership NHS Foundation Trust

Stockport NHS Foundation Trust

Tameside Hospital NHS Foundation Trust

The Tavistock and Portman NHS Foundation Trust

Trafford Healthcare NHS Trust

University College London Hospitals NHS Foundation Trust

University Hospital of South Manchester NHS Foundation Trust

Wakefield District Primary Care Trust

Warrington and Halton Hospitals NHS Foundation Trust

Whittington Health NHS

Wrightington, Wigan and Leigh NHS Foundation Trust

York Teaching Hospital NHS Foundation Trust

### Statistical considerations

#### Power calculation/sample size consideration

The power calculation was based on a minimally important reduction in 18-month repetition rates of self-harm (leading to hospital attendance) from 29 % in participants receiving TAU [48] to 18.8 % in participants receiving FT, that is, a reduction of 35 %. Using a 5 % significance level log-rank test for equality of survival curves, 374 participants per arm were required, with 172 total events, to give 90 % power to detect this reduction in 18 month repetition rates, providing a constant hazard ratio of 1.64. Assuming at most a 10 % loss to follow-up by 18 months for the primary outcome, the total sample size required was 416 per arm, 832 in total.

Although SHIFT is an individually randomised trial, inherent clustering within the data structure (participants nested within therapists) is known to have an impact on power and is related to the level of the intra-cluster correlation coefficient (ICC) and the cluster size. It was expected that the level of clustering would be low - possibly around 0.01 but no higher than 0.05 (due to use of therapy manuals, therapist selection, training, supervision and monitoring). In addition, the number of participants per therapist was expected to be small. In the TAU arm, it was estimated that there would be between eight and 15 therapists available in the team at any one centre, so across the anticipated 15 participating Trusts there would be 120 to 225 therapists available to treat 416 participants. Thus, each therapist would treat between two and four participants (maximum control cluster size of 4). In the FT arm, we estimated that there would be approximately 35 therapists available across all the sites to treat 416 participants; operating in teams of three or four therapists. Within each FT team, each therapist would be the lead for a subset of participants at that site (the other therapists in the team would act as observers and make only a small face to face contribution for those participants). Thus, each FT therapist would have direct contact with approximately 12 participants (maximum intervention cluster size of 12).

The design effect, describing the extent to which the sample size must be increased to obtain the same power as an individually randomised study without clustering, was therefore assumed likely to be no greater than 1.55 (assuming an ICC of 0.05), effectively reducing the sample size from 416 per group to 270 per group and the power from 90 % to around 75 %. If the ICC were as low as 0.01, then the design effect would be 1.11, reducing the sample size to 374 per group and the power to around 85 %. We anticipated the ICC would be towards the lower end of the possible range, and therefore, the trial would still be adequately powered with the sample size planned.

### Statistical analysis

A single formal interim analysis was planned on the primary endpoint, repetition of self-harm leading to hospital attendance within 18 months of randomisation, when at least half the required number of events had been reached (86 events). The DMEC, in light of the interim data and of any advice or evidence they wished to request, would if necessary report to the Trial Steering Committee with a recommendation of trial adaptation or early closure if, compared with TAU, the effect of FT was significantly inferior (*P* < 0.005). Final analyses are not planned until all participants have reached the end of the follow-up period, when it is expected that at least 172 events will have occurred.

All analyses, unless otherwise specified, will be conducted on the intention-to-treat population defined as all participants randomised regardless of non-compliance with the intervention. An overall two-sided 5 % significance level will be used for all endpoint comparisons. For the primary endpoint, this will be adjusted to account for the planned interim analysis. The O’Brien and Fleming alpha spending function will be used [49], allowing an alpha level of 0.047 for the final analysis and 0.005 for the interim analysis.

### Primary endpoint analysis

Cox’s Proportional Hazards Model accounting for the minimisation factors will be used to test for differences in 18-month repetition rates. Hazard Ratios and corresponding 95 % confidence intervals (CIs) will be presented. If a participant is lost to follow-up, they will be treated as censored. Kaplan-Meier curves will be constructed for each group and compared using a two-sided log-rank test. Although the significance level has been slightly reduced to account for an interim analysis, CIs will still be presented at the 95 % level. The extent of clustering on participant outcomes due to therapists and the impact on the precision of the treatment effect estimate will be investigated using multilevel survival frailty models in a secondary analysis. Sensitivity analysis will also be used to assess the impact of missing details of hospital attendance should they remain by the time of analysis (that is, where it is unclear whether an attendance was due to self-harm or not).

### Secondary endpoint analysis

The analysis of 12-month repetition rates will follow that of the 18-month data detailed in the primary endpoint analysis. Further episodes of self-harm will be analysed using a multiple events analysis based on Andersen-Gill [50] methodology, making use of the timing and cumulative number of first and subsequent events. For other measures, such as suicidal ideation and quality of life, mean scores and 95 % CIs (adjusted for baseline) will be presented for each time point, and repeated measures models will be used to estimate differences between the treatment groups. Mediator and moderator variables, which influence engagement with and benefit from treatment, will be identified by modelling the relationship between process variables (for example, number of sessions, medication use, referrals, mediators) and outcomes in a complier average causal effect (CACE) analysis, and via inclusion of potential moderators as an interaction effect in the primary analysis model. In addition, we will summarise adherence and alliance to family therapy.

### Health economic analysis

The economic evaluation will estimate costs of treatments and, if appropriate, the incremental cost-effectiveness of FT compared to TAU in the management of self-harm in adolescents. Two sets of economic evaluation will be undertaken: a set of within-trial analyses and a decision analytic model over a longer time horizon.

### Within-trial economic evaluation analysis

The within-trial evaluation will compare the outcomes and cost up to 18 months follow-up using trial data. In line with the NICE reference case [51], the primary outcome will be quality-adjusted life years (QALYs). The combination of answers to the EQ-5D will lead to a health profile of five digits [52], which will be converted into utility using standard UK tariff values [53]. These utilities will represent patients’ overall quality of life and will be multiplied by the time spent in each state to generate QALYs. Costs will include any resource usage incurred to the NHS and Social Services while providing FT or TAU - as well as records of hospital attendance, and of primary, community or social care service attendance, and medications reported in the health economics questionnaire over the follow-up. Resource usage figures will be converted into costs using unit cost figures from the PSSRU Costs of Health and Social Care [54] and the Department of Health’s Reference Costs [55]. The discount rate will be 3.5 % as per the NICE guidelines [51] and parameter uncertainty will be addressed through probabilistic sensitivity analysis. This analysis will use the non-parametric bootstrap method to generate simulations of the mean costs and effects for each arm of the trial. The same within-trial analysis will be carried out using repetition of self-harm as a secondary outcome.

### Decision analytic model

The decision analysis model will evaluate the cost-effectiveness of FT compared to TAU over a longer time horizon using QALY as the outcome and all health and social care costs. The model will rely on the results of the within-trial analysis and literature searches. Ideally the time horizon of interest would be a lifetime horizon; however, this does not have any precedence in the literature. A cohort of young people will face such a large number of events over their adult life that building a decision analysis model that can reflect real life would be difficult and require would a massive amount of assumption and probabilities to estimate. It will be more realistic to consider shorter time horizons.

We will initially consider extrapolating the cost-effectiveness of FT compared to TAU in the management of self-harm in adolescents up to 5 years after they were randomised in the trial. If we find appropriate literature references from which we can make reliable assumptions and extract appropriate probabilities, we will additionally consider modelling the cost-effectiveness up to the time when the adolescent enters adulthood (20 years old). We will design a Markov model with three health states: stop self-harm, repeat self-harm and death. Depending on the findings from the within-trial analysis and the time to repeat self-harm, the model will be designed to run every month or every 6 months to take account of any incidences in relation to self-harm. Parameter uncertainty will be addressed through probabilistic sensitivity analysis using Monte Carlo simulation.

The outputs of both the within-trial analysis and the decision analysis model will be presented as the expected incremental cost-effectiveness ratios of FT compared to TAU, scatter plots on the cost-effectiveness plane and cost-effectiveness acceptability curves. The expected net benefit of FT compared to TAU will be presented assuming a threshold value of £20 k to £30 k per QALY. Further sensitivity analyses for both analyses will consider additional QALY gains to the caregiver using their answers to the HUI3 questionnaire and additional costs from societal perspective including out-of-pocket expenses, productivity costs to the patients and carers and use of education and justice services.

## Discussion

The project was funded by the NIHR Health Technology Assessment (HTA) Programme in response to a commissioned call. The HTA had identified a gap in the evidence relating to the most appropriate treatment to recommend following self-harm in adolescents. The HTA were very specific that a randomised controlled trial of family therapy versus conventional care was needed for young people, aged 11 to 17, who had self-harmed on more than one occasion.

### Design issues

During the design phase several methodological complexities were encountered, each requiring careful consideration. The delivery of behavioural/psychological interventions, as in SHIFT, involves a number of different therapists with varying experience, hence our first challenge was to choose an appropriate method of randomisation and determine strategies for minimising therapist variation in the FT arm. Using standardised training and supervision processes, we can measure and describe the effect of therapists on outcomes and ensure that the validity of the trial is optimised. As dropout from treatment and follow-up is common following self-harm in clinical services, and also in trials of this nature, the choice of primary outcome is key. Our second challenge was therefore to choose an endpoint that would minimise the loss-to-follow-up rate and be an objective and quantifiable outcome; using hospital attendance data, especially from a central routine data source (HSCIC), maximises accurate and reliable data collection, with secondary outcomes collecting self-reported self-harm to account for self-harm that does not result in hospital attendance. Safety reporting is well established for trials of pharmacological interventions, but this is not the case for trials of behavioural or psychological interventions. Consequently, to ensure adequate safety reporting, we needed to define clearly the expected serious and non-serious adverse events, taking into account the characteristics of the population under investigation. An independent data monitoring and ethics committee was convened to review the safety of the trial during recruitment and follow-up, and a formal interim analysis of the primary endpoint was undertaken to review the safety of family therapy. Finally, a further complexity during the design of SHIFT was the consideration of appropriate analysis techniques. As trial designs become more complicated, so does the analysis, especially when different therapists are involved in delivering the interventions, or when multiple events can be observed such as in SHIFT. Cox’s Proportional Hazards model will be used to test for differences in repetition rates whilst secondary analyses will examine the robustness of the conclusions from the primary analysis. Multiple events analysis will make use of the timing and number of first and subsequent self-harm events, and multilevel survival frailty models will determine the extent of clustering on outcome due to therapists and the impact on the precision of the treatment effect.

### Clinical service engagement

During the early stages of implementation of the trial CAMH Services were slow to engage with SHIFT. We deemed the slow initial recruitment a consequence of clinical unfamiliarity with research in many sites, the pressures of routine clinical practice and the sheer size of the task of involving so many clinicians in trial processes. The nature of the trial was such that anyone within the clinical team undertaking referral assessments could consider a young person’s eligibility for the trial and then be involved in TAU delivery. Ensuring all clinicians were fully informed about trial processes and had the research materials available was logistically complex. Very regular contact between the trial team and local services facilitated a collaborative and flexible approach, and was helped by the appointment of local trial ‘champions’ who took a lead in reminding their local colleagues of trial procedures. This process took the form of newsletters circulated by CTRU, posters for staff rooms and formal presentations delivered at research or team events. In addition, a training DVD was produced in which the Chief Investigator, researchers and trial therapists discussed the salient points of the trial in a short explanatory piece designed for clinicians.

Data collection from a large number of clinicians was not straightforward as there was little opportunity for learning on the clinicians’ part, a TAU clinician might only see one or two SHIFT participants over the duration of the trial so there is opportunity for missing data. Data collection (from clinicians) was thus a labour intensive task, and was ultimately superseded by data collection by researchers (where they could be un-blinded – for example, at the end of follow-up) and by Clinical Research Networks’ Clinical Studies Officers.

Despite the complexity of SHIFT, the trial ultimately recruited well, following implementation of intensive measures to improve screening and recruitment in 2010 after a slow start in 2009, and was able to complete recruitment at the end of 2013 following an extension award by HTA.

The main results relating to the primary outcome will inform treatment of those referred to CAMHS following self-harm via a definitive recommendation regarding the effectiveness of the SHIFT Family Therapy approach. There is also the opportunity for much sub-study work looking in more detail at the secondary outcomes and investigation into the mediating and moderating factors influencing therapeutic outcome, as well as into the therapeutic process itself.

## Trial status

The Data Monitoring and Ethics Committee met in July 2013 to review the safety of family therapy in a formal interim analysis of the primary endpoint, at which point no concerns were raised, and it was recommended that SHIFT continue to recruit the full sample. SHIFT completed recruitment following the randomisation of 832 young people in December 2013. SHIFT is now nearing the end of the 18-month follow-up period for all participants. The HTA monograph will be submitted in April 2016.

## Abbreviations

A & E: Accident and Emergency Department
CAMHS: Child and Adolescent Mental Health Services
CI: confidence interval
CSO: Clinical Studies Officer
CTRU: Clinical Trials Research Unit
DMEC: Data Monitoring and Ethics Committee
FT: family therapy
HES: Hospital Episode Statistics
HSCIC: Health and Social Care Information Centre
HTA: Health Technology Assessment
ICC: intra-cluster correlation coefficient
ICU: Inventory of Callous Unemotional Traits
LFTRC: Leeds Family Therapy Research Centre
MRC: Medical Research Council
NHS: National Health Service
NICE: National Institute for Health and Care Excellence
NIHR: National Institute for Health Research
QUALY: quality adjusted life years
REC: Research Ethics Committee
SOFTA: System for Observing Family Therapy Alliances
TAU: treatment as usual
UKCP: United Kingdom Council for Psychotherapy
